# Minimally Invasive Drainage for Diabetic Foot Phlegmon †

**DOI:** 10.3390/jcm14113918

**Published:** 2025-06-03

**Authors:** Marco Cavallini

**Affiliations:** Department of Medical-Surgical Sciences and Translational Medicine, AOU Sant’Andrea, Sapienza, University of Rome, 00189 Rome, Italy; marco.cavallini@uniroma1.it

**Keywords:** diabetic foot ulcer, neuro-ischemic foot, surgical drainage

## Abstract

Treatment for deep diabetic foot ulcers (DFUs) and infections (DFIs) includes debridement of any dead/infected non-viable tissue, systemic antibiotics, and surgical drainage to avoid exudate stasis. Surgical drainage can cause extended incisions leading to long scars which expose these sites to ulcer recurrence and inadequate rehabilitation. In order to treat the negative impact of stasis on wound healing, we have designed an easy, minimally invasive surgical drainage technique which allows adequate ulcer cleansing by daily irrigation of any drained tract. A probe is passed along the ulcer’s infected recesses until the end and pushed against the skin, which is incised and pierced. A small 6 Fr-size silastic tube is then anchored to the probe and pulled backwards. The two ends of the tube are tied together to construct an ulcer-piercing drainage (UPD) ring. The UPD ring is designed to keep any tract open for irrigation with a syringe through both sides of the skin opening. The UPD procedure is easy and safe. The constructed blocked ring of tubing the system avoids the possibility for drainage displacement or accidental removal and can be easily utilized by any home caregiver. The UPD and irrigation are useful to provide any recess cleansing, reduce critical and negative ulcer bioburden and bacterial load, and it could avoid unnecessary and untimely extended surgical incisions leading to long uncomfortable scars, inadequate rehabilitation, relapses, or distal amputations.

## 1. Introduction

The pathophysiology of DFU disease is complex and several factors may play a role in the development of foot ulcers, such as the presence of diabetic polyneuropathy and/or peripheral arterial disease (PAD). Clinical and instrumental evidence of PAD is documented in about 50% of patients with a DFU [1]. Moreover, these ulcers may become deeper and infected. Therefore, the population of individuals presenting with DFUs is relatively heterogeneous.

DFI patients have reported a complex interplay of several inflammatory markers which can affect the cardio-vascular system and metabolic homeostasis [2] and DFI may lead to faster progression of cardio-vascular damage and morbidity [3,4]. DFU healing has been documented as an independent factor predicting life expectancy and a longer amputation-free interval [2]. It has been estimated that DFUs precede an estimated 80% of diabetic lower-extremity amputations [5].

Therefore, not only do DFUs require early management, but they also require the assessment of all comorbidities that may influence the outcomes.

Deep DFI must be considered as a surgical infection where antibiotics alone are not sufficient and synergy with an adequate and effective surgical treatment is required.

**Figure 1:**
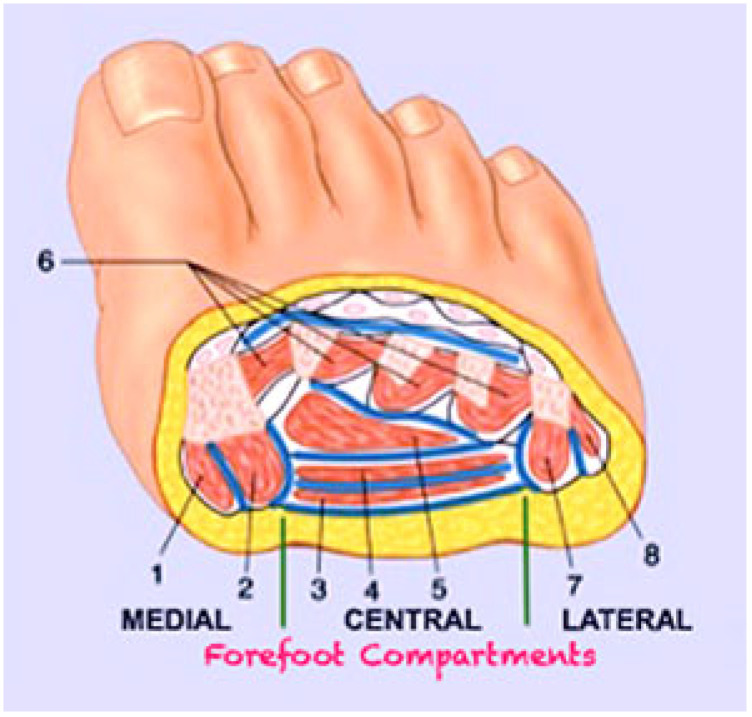
Schematic representation of the 4 main deep spaces of the foot at the level of a transmetatarsal section: medial plantar, central plantar, lateral plantar, and dorsal. Within these virtual spaces, it is possible to identify (blue lines) the diffusion and collection pathways of the exudate to form internal phlegmons, two in the medial compartment, four in the central compartment, and two in the lateral compartment. At the dorsal level, since there are no longitudinal septa, only one large subfascial space is identified. The main muscle groups involved in this stratification are 1: abductor of the 1st toe, 2: flexor brevis of the 1st toe, 3: flexor brevis of the toes. 4: long toes flexor, 5: adductor of the 1st toe, 6: interosseous, 7: flexor of the 5th toe, and 8: abductor of the 5th toe.

DFI often results, as a consequence of the neuropathic unsensitive foot, in a deep soft-tissue phlegmon, which is a progression of the exudate and infection in the underlying tissues with no limits, more frequently involving tendons and forefoot phalanx or metatarsal bones, and which could spread proximally involving the plantar and leg compartments (Figure 1).

When dealing with deep DFI, the primary principle of treating a surgical infection is source control. According to T.I.M.E. (tissue, infection, moisture, and edge) procedure for infected DFU treatment, source control includes resecting or debriding any dead/infected/non-viable soft tissue and avoiding fluid stasis by draining any recess. Surgical drainage is mandatory for the prevention of any fluid or exudate stasis. Stasis is the main thing responsible for persistent bacterial contamination and load, host reaction, and wound healing impairment and delay. Galen of Pergamum (131–201 AD), an ancient Roman gladiators’ physician who was an expert in treating infected wounds, stated “ubi pus ibi evacua” (where there is pus take it out) [6].

In cases of a deep infected ulcer recess or fistula, up to now surgical treatment and drainage has mainly consisted of extended incisions and wide opening of the tract or recess and of the overlying tissues, followed by the removal of all infected non-viable soft tissues and by the apposition of a draining gauze or rubber drain. This approach often results in a large and uncomfortable incision scar, exposing that site to recurrent complications, relapse, and/or inadequate rehabilitation. Moreover, in the case of a neuro-ischemic DFU, these extended incisions involve suffering, non-well perfused tissues, and can be responsible for perilesional tissue damage. In order to treat the negative impact of stasis on wound healing and to avoid undue and untimely initial excessive tissue damage with extended or minor amputation surgical procedures, we have originally designed and pioneered [7,8,9] a minimally invasive and easy surgical approach of DFU drainage, named ulcer-piercing drainage (UPD), which allows an adequate cleansing of the ulcer recess and facilitates the irrigation of any pierced hidden tract.

## 2. Surgical Technique

Following the initial ground-breaking publications on the UPD technique [7,8,9], the latest updated publication, as an abstract, has been presented in the Poster section at the EWMA 2020 Conference [10]. UPD has been utilized in a continuous series of 57 DFIs of selected diabetic patients affected by a Wagner stage 3 (Texas University grade 3–5 and stage B/D) deep skin ulcers of a toe (n = 26), metatarsal forefoot (n = 24), and plantar Charcot foot (n = 7) and with an adequate foot arterial blood supply. The limb’s arterial blood supply was considered as clinically adequate in the presence of peripheral (tibial) arterial pulses, skin and nail trophism, or assessed instrumentally with an ABI > 0.6 and/or a TcPO_2_ > 30 mmHg. A total of 35 DFUs (61%) have been classified as neuro-ischemic (PAD + neuropathy) and 8/35 (23%) have been successfully revascularized by endovascular angioplasty. All patients have been treated with systemic-specific antibiotics on the basis of the results of the ulcer bed culture and relative antibiotic assay [10].

**Figure 2:**
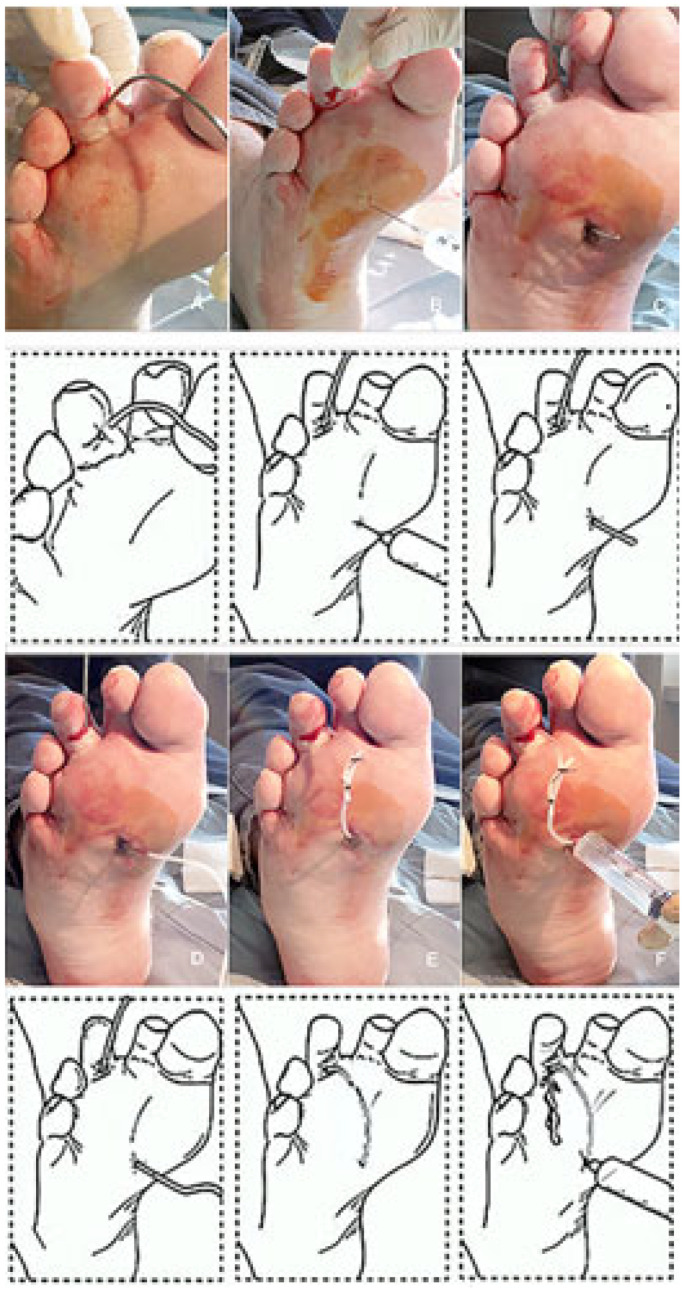
Phases for the construction of the ulcer piercing drainage (UPD) (see text).

Starting from the ulcer bed, with a blunt probe it is possible to follow any narrow, long, and winding recess created by the phlegmon (Figure 2).

**Figure 3:**
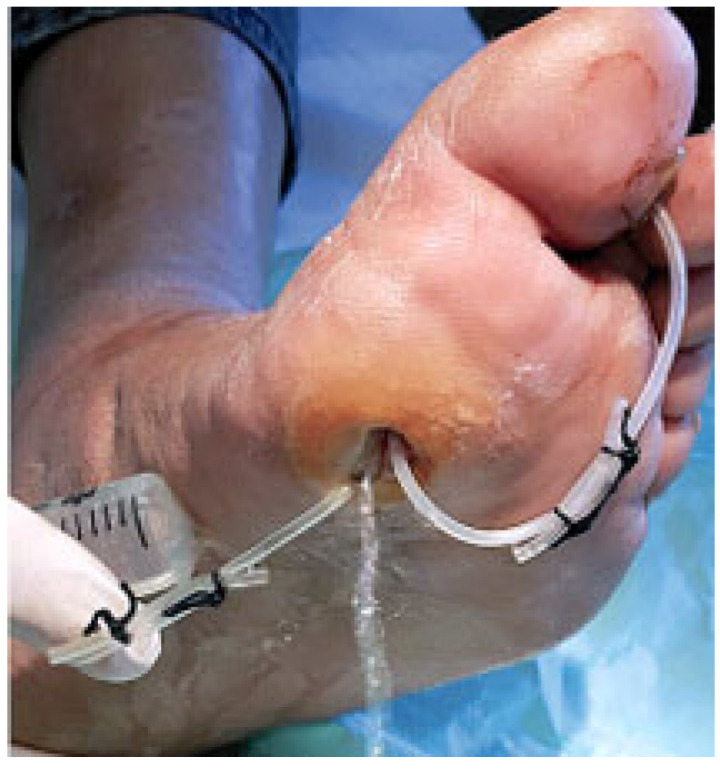
The UP ring is designed to keep any tract open and to facilitate the insertion of a syringe into both sides of the opening to facilitate the irrigation of the drained recess.

At the opposite side and at the end of the phlegmon tract the probe is pushed toward the overlying fascia and skin (Figure 2A). After local anesthesia, the interposed tissues and the skin are pierced and incised in order to pass through the probe (Figure 2B,C). A silicon butterfly-needle tube is, thereafter, anchored to the probe (Figure 2D) in order to pass it backwards along the phlegmon tract. Once this thin tube is passed along the ulcer’s recess tract, the two ends are tied together with two silk stitches in order to construct a UPD ring (Figure 2E). The UPD ring, therefore, is designed to keep the tract open and to facilitate the insertion of a syringe into both sides of the opening (Figure 2F) and facilitate the irrigation of the drained recess (Figure 3). Fistula tract irrigation is than scheduled once or twice a day with 20–40 mL of Dakin’s solution (or other oxidizing solution or crystalloids). The drainage is scheduled to be changed every 2–3 weeks or removed when the growth of the granulation tissue around the tube is closer to the drainage.

**Figure 4:**
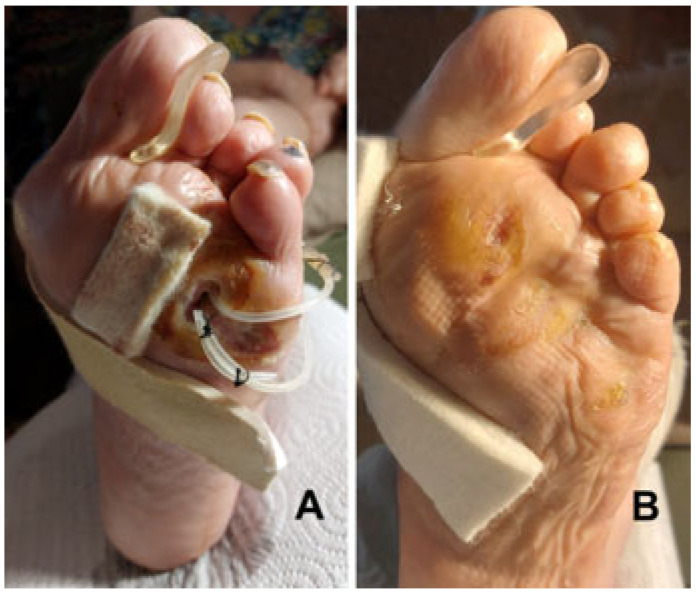
(**A**) After the removal of the 4th and 5th metatarsal heads through the bottom of the deep plantar ulcer, two UPD drains are positioned in dorsal counter-opening to allow daily irrigation of the residual cavities. (**B**) Four months later the ulcers have healed with the realignment of the rays.

A total of 55/57 (96%) ulcers healed within 9 months. In 5 cases (5/26, 19%) this was after toe amputation because of a resulting ischemic toe, whilst 2 cases of acute Charcot foot underwent BKA due to subsequent untreatable foot instability, but after better local control of tissue infection. In 18 DFIs with metatarsal forefoot plantar ulcers (18/24, 75%), the amputation of the infected metatarsal heads has been performed throughout the ulcer bed (Figure 4).

## 3. Discussion

Inflammation is a physiological response to wounding and represents, after an acute injury, the early phase of wound healing. Excessive inflammation, due to the persistence of a critical bacterial contamination, biofilm, or infection, leads to wound chronicity [11]. Stalling of ulcer healing, indicated by those which do not progress beyond the inflammatory phase, has been related to persistent inflammation [12,13] and an increase in matrix metalloproteases, elastase, and hyaluronidase activities [14,15]. Chronic degradation of the extracellular matrix, an increase in proinflammatory cytokines (TNF-α, IL-1, IL-6) [16] which inhibit downregulation of the immune response [17], and soft tissue edema interfering with an already impaired underlying PAD all further hinder wound healing. Other conditions such as an alkaline pH [18] and the high concentration of reactive oxidant substances (ROS), leading to oxidative stress, are part of this complex negative ulcer bioburden [19,20]. Therefore, any local or systemic treatment finalized to eliminate or reduce bacterial load and prolonged inflammation revitalizes physiological tissue healing, reduces exudate and edema, and is associated with a reduction in those critical and negative conditions.

On this topic, some authors have underlined [21] the effectiveness of careful surgical debridement and subsequent local negative pressure therapy in improving and accelerating ulcer healing. More specifically, local negative pressure therapy results in a continuous aspiration of the ulcer bed; therefore, reducing the possibility of exudate stasis, tissue edema, and critical bacterial concentration. This procedure is particularly useful and effective in patients with a deep DFU [22,23]. If the goal is to reduce the bacterial load to a level that does not stimulate the host reaction, and if this can be achieved via daily negative pressure, similar results could be obtained with positive pressure irrigation. This approach has been reported to be particularly useful in cases with a long, narrow, and winding recess, as we often see in cases with phlegmons of the diabetic foot where negative pressure could not be adequate and effective [8,9,10]. Therefore, local negative pressure with daily aspiration and local positive pressure with irrigation can represent two sides of the same coin, which is to avoid exudate stasis and reduce bacterial load.

**Figure 5:**
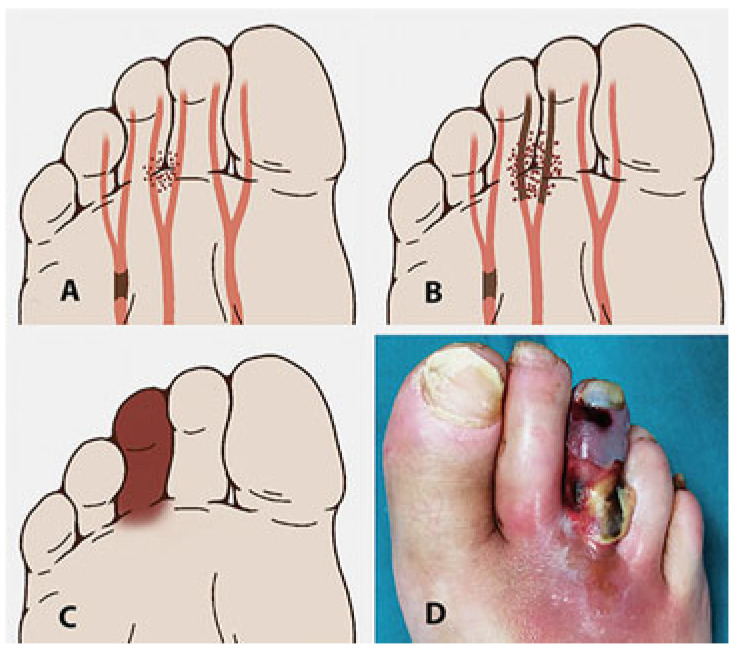
In the case of a neuro-ischemic foot, PAD is associated with adequate tissue vascularization (**A**). The association and presence of a septic focus (black dots) can cause tissue oxygenation to precipitate into a locally critical condition caused by tissue edema, endothelial damage, and/or thrombosis of toes’ terminal arterioles within a toxic bioburden (**B**) leading to tissue necrosis (infectious gangrene) (**C**,**D**).

When dealing with neuro-ischemic DFI with an underlying non-critical PAD and infection (Figure 5), the odds ratio of nonhealing is tripled versus DFU lesions with these negative underlying conditions alone [1].

**Figure 6:**
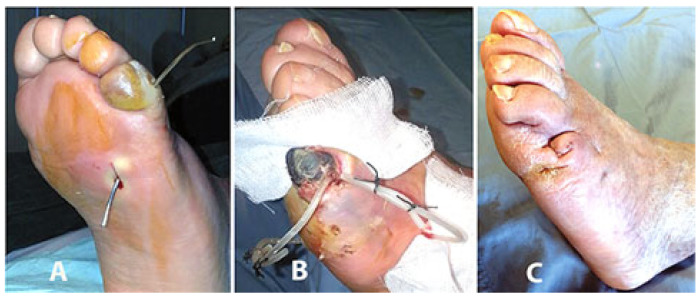
Infectious gangrene of the 5th ray of a neuro-ischemic foot with non-critical PAD, with osteomyelitis of the proximal phalanx and phlegmon extending plantarly and dorsally (**A**) and cleared and drained with a minimally invasive technique (**B**). Healing was achieved in 5 months after the amputation of the necrotic fifth toe and with minimal scar tissue (**C**).

The downgrading of this negative clinical picture could be achieved with a reduction in the infection and local bacterial load, bioburden, and inflammatory edema, which allows for the improvement of local tissue perfusion and enhances tissue repair. If wound healing is improved with the appearance of stable granulation tissue it is often possible to avoid or delay untimely revascularization procedures. Indeed, the acute inflammatory state of neuro-ischemic foot tissues requires less traumatic and minimally invasive surgical management since excessive trauma can result in tissue damage while wider surgical incisions may interfere with an already critical vascular network and deficient tissue perfusion (Figure 6).

**Figure 7:**
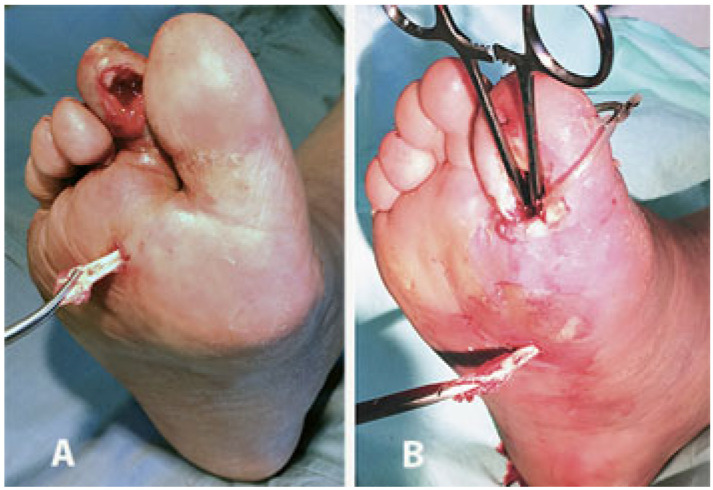
Radical surgical debridement can include the removal of bone and tendon infected tissues; (**A**) removal of the 2nd toe infected flexor tendon; (**B**) removal of the infected flexor tendon of the 1st toe already drained with a dorsal counter-incision and with the debridement of the infected proximal phalanx. In this case, the redness of the plantar midfoot indicates that the phlegmon is extended to that level along with flexor tendon.

**Figure 8:**
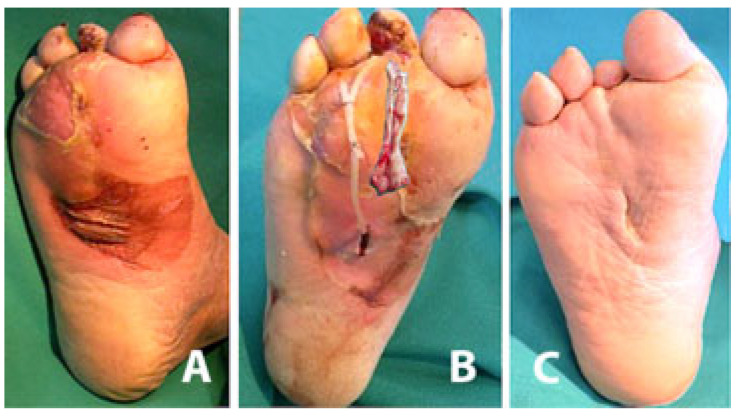
(**A**) Phlegmon of the right foot arising from a 2nd toe ulcer and spreading along the plantar fascia. (**B**) UP for drainage and irrigation of the recess and the infected flexor tendon (shown) removed from the distal incision which has been slightly extended. (**C**) Healing with small scars occurred after about 7 months. The resulting scar is in a plantar area less exposed to pressure, overload, mechanical stresses, and risk of relapse.

Once the acute and more critical inflammatory phase is drained and under better control, to achieve ulcer healing in patients with adequate tissue arterial blood supply surgical debridement of the ulcer bed and removal of all infected/instable/dead non-viable soft tissues are mandatory, along with further drainage of all ulcer recesses or fistulas and systemic antibiotics (Figure 7 and Figure 8).

Up to now, in cases of a deep narrow ulcer recess, surgical drainage consists of extended incisions of the overlying tissues of the infected tract and of those in apposition to a draining gauze or rubber drain. This solution, however, is not always effective, safe, and tissue sparing, and it could be detrimental since those voluminous, often not well-fixed drainages can become obstructive or can be easily and/or accidentally removed during homecare medication. Moreover, any skin wound, fistula, or incision progressively reduces its opening, therefore interfering with ulcer tract drainage.

**Figure 9:**
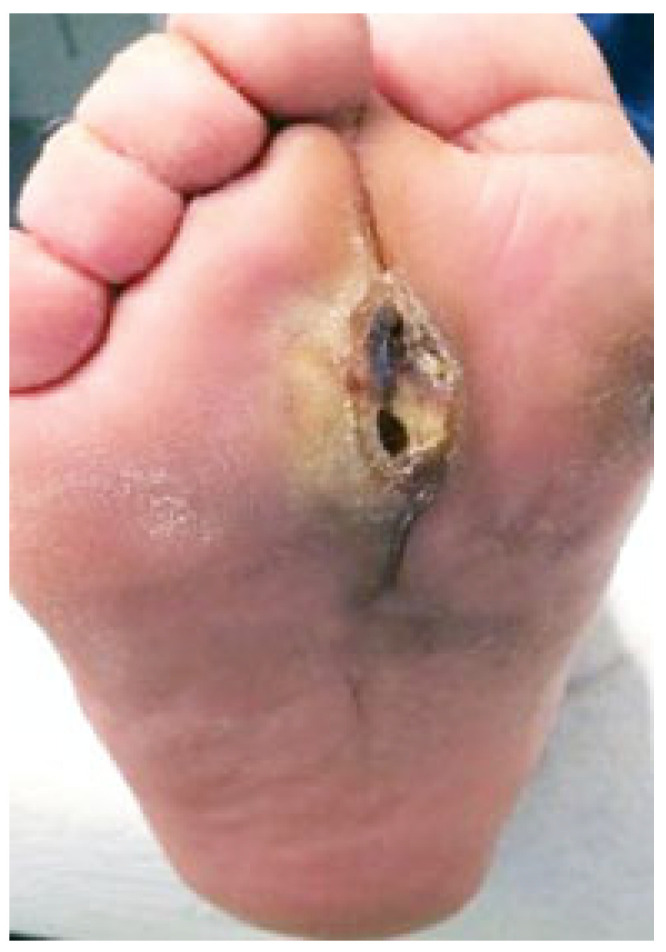
Relapse of a plantar DFU previously treated with an extended surgical incision, resulting in a long plantar scar exposed to pressure, overload, mechanical stresses, and relapse.

Minimally invasive UPD approach is less traumatic, more tissue sparing, and is particularly indicated in those patients where an insufficient, while compensated, lower limb PAD is often underlying the complicated DFU. Moreover, the UPD approach avoids untimely and often unnecessary wider tissue incisions and scarring which, especially in cases of surfaces exposed to pressure, overload, and mechanical stresses, could be responsible for relapses. Indeed, the interface between elastic normal tissue and the scar anelastic fibrotic tissue is exposed to small deep hematomas and, thereafter, to bacterial contamination, deep infection, and ulcer relapse [24] (Figure 9).

**Figure 10:**
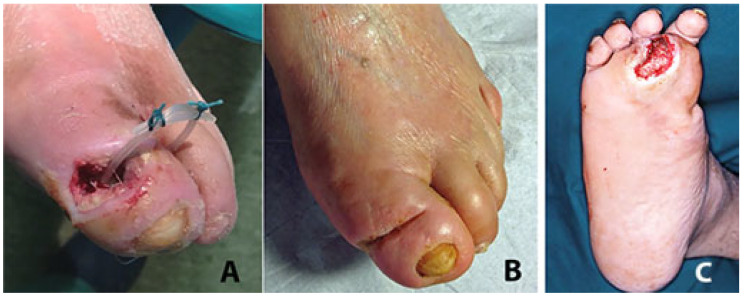
UP drainage (**A**) in the case of a DFU with osteomyelitis of both phalanxes of first toe in a diabetic patient with PAD and chronic respiratory failure, healed (**B**) after 5 months. (**C**) Any therapeutic attempt must be explored to avoid amputation of the first ray, because mid-forefoot plantar ulcer due to overload is a consequence of first ray resection.

**Figure 11:**
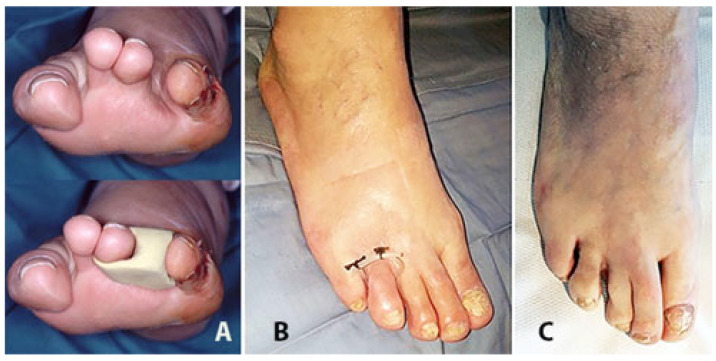
Following 4th toe amputation to avoid displacement of the remaining toes a silicon orthesis is needed (**A**). Surgical conservative approach of phalanx osteomyelitis with a UP drainage and infected bone removal can avoid this critical condition (**B**,**C**).

**Figure 12:**
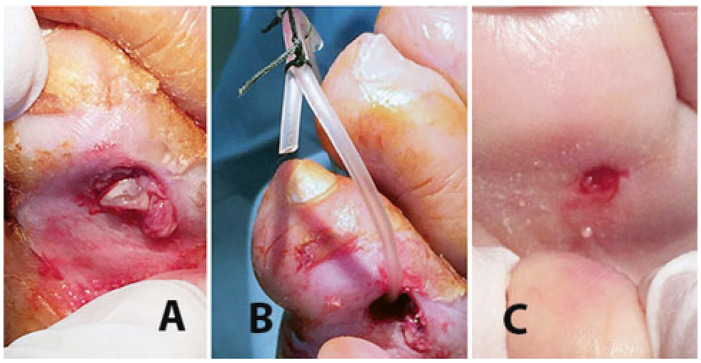
The exposed infected phalanx of the 4th toe (**A**) has been removed, and the residual cavity (**B**) was drained with a UPD through a medial counterincision (**B**) to achieve healing after about 3 months (**C**).

Despite the concept that less-is-more and the need for a tissue sparing approach in treating DFIs, diabetic foot minor amputations are reported in about 18% of these diabetic patients [25]. UPD is a minimally invasive and conservative technique that, when applied to an infected toe which often has a contaminated exposed bone, further avoids the following two main complications of toe amputation: (a) in case of the amputation of the first ray, the development of a mid-forefoot plantar ulcer due to an overload of that area (Figure 10) and (b) in case of a middle toe amputation, the resulting wider interdigital space which exposes a forefoot deformity and that can be treated only with the interposition of a silicon digital orthosis (Figure 11 and Figure 12).

**Figure 13:**
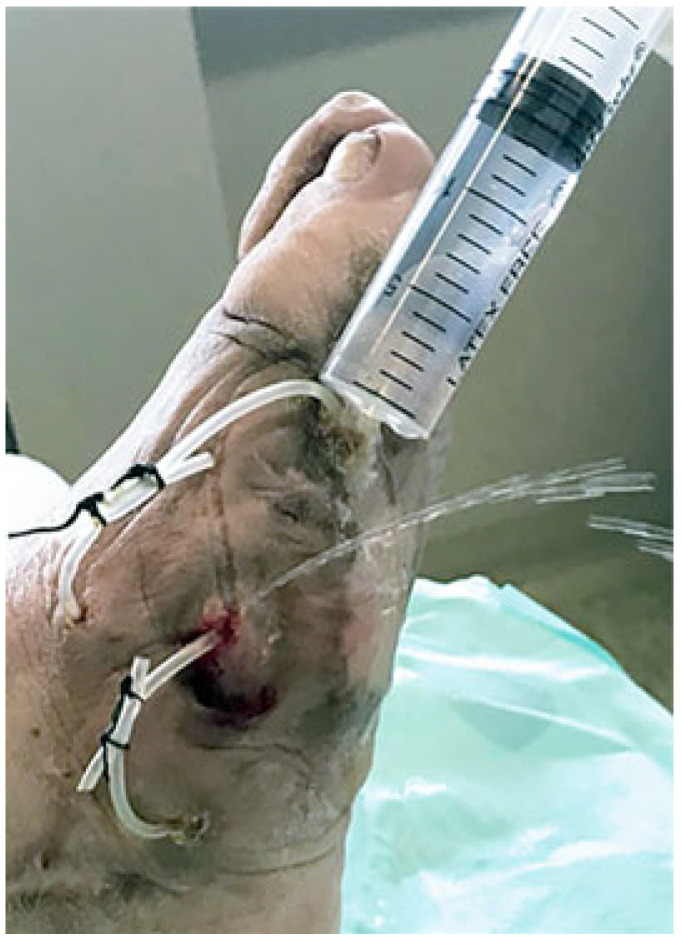
Pressure irrigation of two recess-infected tracts drained with two UPD rings.

In conclusion, in the case of a deep and narrow not-well-drained ulcer recess, the UPD procedure, in our opinion, is useful to ensure effective tract cleansing and reduce bacterial load by daily positive pressure irrigation (Figure 13).

**Figure 14:**
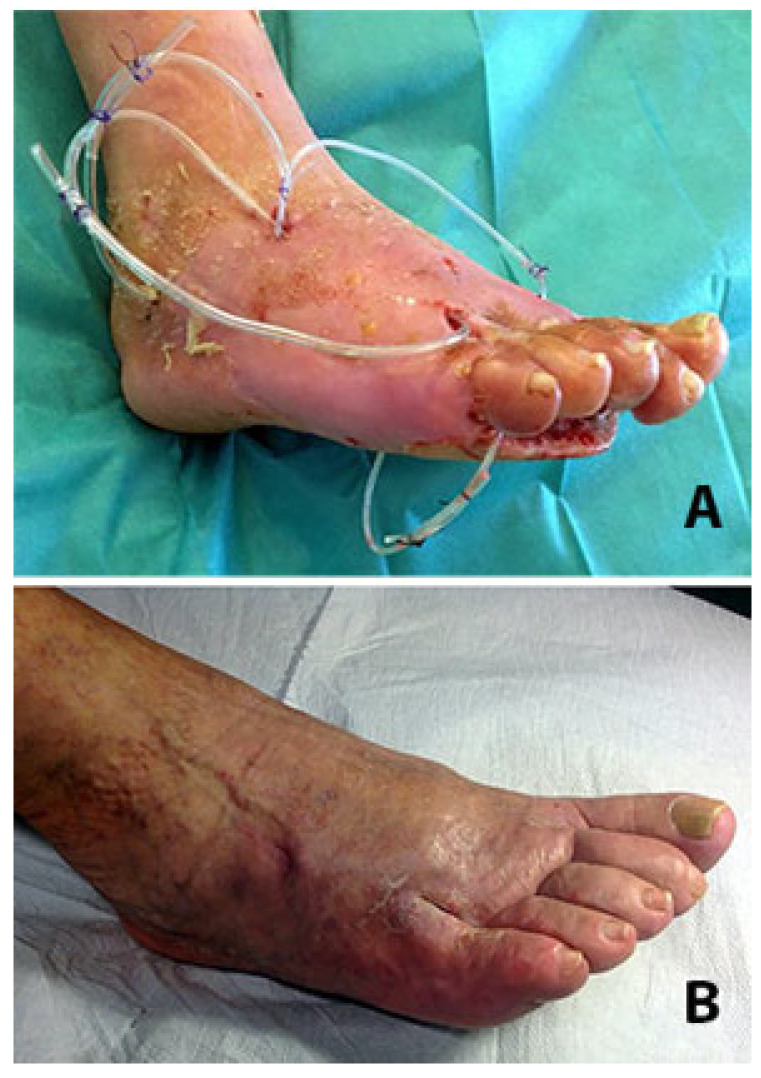
(**A**) Multiple dorsal and plantar UP drainages to treat extended phlegmons with multiple recesses. (**B**) Complete healing has been observed after 4 months.

The UPD procedure is easy and safe and the constructed blocked ring of the small-size silicon tube system avoids the possibility of drainage displacement, accidental removal, or recess obstruction. Moreover, this procedure increases the ease at which the patients themselves or the caregivers (nurses or family) can perform daily cleansing and wound care in the homecare setting. Finally, this conservative, minimally invasive technique should be considered because it could avoid unnecessary and often untimely extended and deep tissue incisions which, as a consequence, can result in scarring and less tissue sparing. This is emphasized by the fact that scarring could interfere with rehabilitation and could be responsible for ulcer recurrence (Figure 14).

The minimally invasive procedure of UPD represents a small but effective step towards a less traumatic and conservative treatment of deep cutaneous ulcers with all not-well-drained recesses.

In diabetic patients with deep DFUs complicated by infected recesses, we consider UPD as a first surgical option to treat DFI phlegmons. Additionally, it does not interfere with other further and more invasive surgical procedures, if needed.
